# Interaction of Age and Self-reported Physical Sports Activity on White Matter Hyperintensity Volume in Healthy Older Adults

**DOI:** 10.3389/fnagi.2020.576025

**Published:** 2020-11-02

**Authors:** Mary Kathryn Franchetti, Pradyumna K. Bharadwaj, Lauren A. Nguyen, Emily J. Van Etten, Yann C. Klimentidis, Georg A. Hishaw, Theodore P. Trouard, David A. Raichlen, Gene E. Alexander

**Affiliations:** ^1^Department of Psychology, University of Arizona, Tucson, AZ, United States; ^2^Evelyn F. McKnight Brain Institute, Tucson, AZ, United States; ^3^Arizona Alzheimer’s Consortium, Phoenix, AZ, United States; ^4^Department of Epidemiology and Biostatistics, University of Arizona, Tucson, AZ, United States; ^5^Department of Neurology, University of Arizona, Tucson, AZ, United States; ^6^Department of Biomedical Engineering, University of Arizona, Tucson, AZ, United States; ^7^Department of Medical Imaging, University of Arizona, Tucson, AZ, United States; ^8^Human and Evolutionary Biology Section, Department of Biological Sciences, University of Southern California, Los Angeles, CA, United States; ^9^Neuroscience Graduate Interdisciplinary Program, University of Arizona, Tucson, AZ, United States; ^10^Physiological Sciences Graduate Interdisciplinary Program, University of Arizona, Tucson, AZ, United States; ^11^Department of Psychiatry, University of Arizona, Tucson, AZ, United States

**Keywords:** physical activity (exercise), moderate to vigorous physical activity (MVPA), MRI, white matter (WM), regional white matter lesion load, white matter hyperintensity volume, healthy aging

## Abstract

Cerebral white matter (WM) lesion load, as measured by white matter hyperintensity (WMH) volume with magnetic resonance imaging (MRI), has been associated with increasing age and cardiovascular risk factors, like hypertension. Physical sports activity (PSA) may play an important role in maintaining WM in the context of healthy aging. In 196 healthy older adults, we investigated whether participants reporting high levels of PSA (*n* = 36) had reduced total and regional WMH volumes compared to those reporting low levels of PSA (*n* = 160). Age group [young-old (YO) = 50–69 years; old-old (OO) = 70–89 years], PSA group, and age by PSA group interaction effects were tested, with sex, hypertension, and body mass index (BMI) as covariates. We found significant main effects for age group and age by PSA group interactions for total, frontal, temporal, and parietal WMH volumes. There were no main effects of PSA group on WMH volumes. The OO group with low PSA had greater total, frontal, temporal, and parietal WMH volumes than the YO with low PSA and OO with high PSA groups. WMH volumes for the YO and OO groups with high PSA were comparable. These findings indicate an age group difference in those with low PSA, with greater WMH volumes in older adults, which was not observed in those with high PSA. The results suggest that engaging in high levels of PSA may be an important lifestyle factor that can help to diminish WMH lesion load in old age, potentially reducing the impact of brain aging.

## Introduction

Healthy aging is associated with increases in white matter hyperintensity (WMH) volumes on magnetic resonance imaging (MRI) that are thought to preferentially impact frontal and deep periventricular brain regions (Holland et al., 2008; Marquine et al., 2010). These regions of WMH reflect macrostructural changes in white matter (WM) lesion load that increases with age (Yoshita et al., 2006) and has been associated with deficits in cognitive performance (Smith et al., 2011). It has been suggested that WMH lesions may be a consequence of chronic ischemia related to cerebral small vessel disease (SVD) with a dose-dependent relationship observed between WMH volumes and poorer clinical outcomes (Prins and Scheltens, 2015). Age-related changes in cardiovascular health related to high blood pressure have been suggested as a primary pathophysiological risk factor, with hypertension being associated with both advanced age and greater WMH severity (Firbank et al., 2007). On the other hand, healthy older adults without hypertension often have WMH lesions, suggesting that such alterations of WM are common in healthy aging, even in the absence of major cardiovascular risk factors (Tseng et al., 2013).

Physical activity (PA) may play an important role in maintaining WM in the context of healthy aging (Torres et al., 2015). Studies in both humans and non-human animal models have highlighted the brain-based benefits of engaging in aerobic PA and exercise pointing to multiple pathways related to increased perfusion, angiogenesis, neurogenesis, synaptogenesis, and myelin remodeling and/or myelination that can support neuroplasticity and help to maintain WM integrity (O’Rourke et al., 2014; Alexander, 2017; Raichlen and Alexander, 2017). In a study of older adult masters athletes, who engaged in life-long exercise training and elite-level competitive sports, Tseng et al. (2013) found that, compared to sedentary elderly, the masters athletes had significantly less deep WMH volumes. Also, a study by Burzynska et al. (2014) evaluated the association of both PA and cardiorespiratory fitness (CRF; VO_2max_; maximum oxygen consumption during exercise) with measures of WM integrity and WMH lesion load in low-fit adults. As measured by accelerometry, greater moderate-to-vigorous PA (MVPA) was associated with reduced WMH volumes and the results were not related to CRF (Burzynska et al., 2014). Furthermore, using a sample of 7,148 middle-aged to older adults ages 45–80 from the UK Biobank, Raichlen et al. (2019) found that both MVPA and CRF were inversely associated with WMH lesion load after controlling for age, sex, and cardiovascular risk factors. Together, these findings suggest that MVPA may have beneficial effects on the aging brain aside from those related to cardiovascular health, including the reduction of WM lesion load (Raichlen et al., 2019). Whether age and PA among older adults interact to influence total and regional WMH volumes in healthy aging, however, has yet to be fully investigated.

Using a self-report PA questionnaire in a sample of 691 community-dwelling adults in their early 70s, Gow et al. (2012) found that PA (including sports activities) was associated with less WM lesion load 3 years later. Also, it has been suggested that reports of engagement in sports activities provide a more reliable self-report measure of PA in older adults (Sylvia et al., 2014). Thus, self-report PA questionnaires that specifically assess daily recreational physical sports activity (PSA) may be especially helpful in evaluating age-related differences in brain structure, including WMH volumes.

For the current study, we sought to investigate the interactive effects of age group and level of self-reported PSA (e.g., running, jogging, cycling, or swimming) on WMH volumes in a cohort of community-dwelling, healthy older adults, ages 50–89 years. We hypothesized that old-old (OO) adults (ages 70–89) with low levels of PSA would have greater total and frontal lobar regional WMH volumes compared to OO adults with high levels of PSA and young-old (YO) adults (ages 50–69) with high or low levels of PSA.

## Materials and Methods

### Participants and Procedures

A sample of 196 adults, ages 50–89 years, were drawn from a longitudinal cohort study of cognitive aging that included 210 healthy older adults. Enrollment criteria and procedures for the cohort have been described (Nguyen et al., 2016; Van Etten et al., 2020). They were community-dwelling older adult volunteers living in the Tucson-metro area and were recruited by newspaper advertisement. The participants in this study were mainly Caucasian (93.88%) with 101 male (51.53%; mean age = 70.13, SD = 1.05) and 95 (48.47%; mean age = 69.37, SD = 1.09) female. The sample had an average of 15.83 years (SD = 2.56) of education and had an average score on the Mini-Mental State Exam (MMSE; Folstein et al., 1975) of 28.96 (SD = 1.24). Fourteen participants were excluded from analyses due to either missing data or poor quality neuroimaging data (e.g., excessive head movement during scanning). The sample was grouped by their median age into YO (ages 50–69; *n* = 98) and OO (ages 70–89; *n* = 98) participants for comparison in relation to levels of PSA, as we expected WMH volumes in the older age group in the cohort to be preferentially impacted by levels of PSA. For the low PSA groups, there were 83 participants in the YO and 77 in the OO groups, whereas, for those with high PSA, there were 15 participants in the YO and 21 in the OO groups. There was no difference in the proportion of the YO and OO participants across the PSA groups ($\chi_{\left(1,N=196\right)}^{2}$ = 1.23, *p* = 0.27).

Before they were enrolled in the study, participants underwent an extensive medical screen to exclude significant neurological and psychiatric disorders. Information on their medical history and medication status were obtained and they had a physical and neurological examination performed by a neurologist (GH) who specializes in age-related cognitive disorders. The participants completed rating scales and questionnaires to assess functional capacity (Lawton and Brody, 1969), family history, quality of sleep (Buysse et al., 1989), and the presence of current symptoms of depression with the Hamilton Depression Rating Scale (HAM-D; Hamilton, 1960). The sample participants were excluded from the study if they had a HAM-D score ≥10 or MMSE score <26. Procedures for this study were approved by the University of Arizona Institutional Review Board and all participants gave their informed written consent for participation.

### Physical Activity Questionnaire

All subjects completed the Physical Activity Questionnaire (PAQ), which provides a self-report measure of household activities, sports activities, and physically active leisure time activities (Voorrips et al., 1991). The PAQ asks participants to self-report their habitual physical activities of the last year (Baecke et al., 1982; Voorrips et al., 1991). Sports activities are reported as a specific type of activity (e.g., running, jogging, cycling, swimming), hours per week engaged in the activity, and period of the year in which the activity is normally performed (Voorrips et al., 1991). An intensity code based on net energetic cost is used to classify each activity (Bink et al., 1966; Voorrips et al., 1991).

For this study, we focused on self-report ratings of sports activity. Given that many sports activities involve moderate-to-vigorous aerobic activity, such as running/jogging, cycling, and swimming, engaging in such activities is more likely to result in sustained moderate and/or vigorous-intensity, compared to lower intensity household and/or leisure activities. Engaging in MVPA is often needed to improve cardiovascular fitness, which is a key physiological factor linking PA to improved cognitive functioning and brain health (Raichlen and Alexander, 2017).

The PAQ was used to categorize participants as being engaged in either high levels of PSA (PAQ ≥ 2) or low levels of PSA (PAQ < 2). A score of ≥2 corresponds to engagement in PSA more than 9 months of the year for approximately 2–3 h per week and includes engaging in sports such as running/jogging, cycling, and swimming. We chose the ≥2 thresholds for high sports activity in our cohort as it is consistent with the recommended 2.5 h of moderately intense weekly activity proposed by the World Health Organization (WHO) to improve cardiorespiratory fitness in older adults (World Health Organization, 2010).

### Image Acquisition

All MRI scans were acquired on a 3T GE Signa scanner using an eight-channel phased array coil (HD Signa Excite, General Electric, Milwaukee, WI, USA). Volumetric T1-weighted Spoiled Gradient Echo (SPGR) MRI scans (slice thickness = 1.0 mm, TR = 5.3 ms, TE = 2.0 ms, TI = 500 ms, flip angle = 15°, matrix = 256 × 256, FOV = 25.6 cm) and T2 Fluid-Attenuated Inversion Recovery (FLAIR) scans (slice thickness = 2.6 mm, TR = 11,000 ms, TE = 120 ms, TI = 2,250 ms, flip angle = 90°, matrix = 256 × 256, FOV = 25.0 cm) were obtained for each participant.

### Image Processing of White Matter Hyperintensity Volume

The volumes of the WMH lesions were computed with a multispectral, automated lesion segmentation toolbox (LST) for Statistical Parametric Mapping (SPM12; Wellcome Trust Centre for Neuroimaging, London, UK) using a combination of the T1-weighted volumetric and T2-FLAIR MRI scans (Schmidt et al., 2012). WMH probability maps were generated by LST at a range of values for the optimization parameter kappa and were inspected to determine an optimal threshold (0.35) for this healthy aging cohort. These WMH probability maps were thresholded at 1 and voxel volumes were summed to determine total WMH volumes in milliliters (ml).

**Figure 1:**
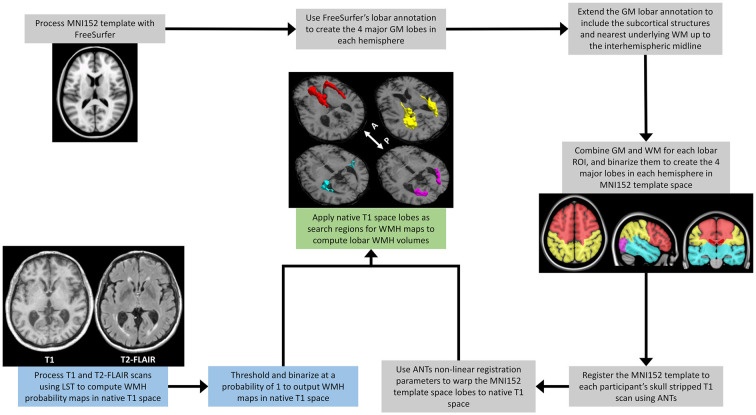
A schematic outline of the image processing steps involved in extracting regional lobar WMH volumes. The process combines outputs from the multispectral LST processing of a participant’s T1-weighted and T2-FLAIR scans (blue text boxes), with those from the FreeSurfer (https://surfer.nmr.mgh.harvard/fswiki) processing of the MNI152 template (gray text boxes), to provide WMH volumes shown for frontal (red), temporal (cyan), parietal (yellow), and occipital (magenta) lobar regions (green text box). LST, Lesion Segmentation Toolbox (Schmidt et al., 2012); WMH, White Matter Hyperintensities; MNI152, The Montreal Neurological Institute template (McConnell Brain Imaging Centre, McGill University) derived from the linear and high-dimensional non-linear registration of 152 T1-weighted structural images into a common average space; GM, Gray Matter; WM, White Matter; ANTs, Advanced Normalization Tools (Avants et al., 2011); White arrow with A and P, anterior and posterior directional orientation of MRI axial brain slices.

The steps for determining the regional lobar WMH volumes are shown in Figure 1. The MNI152 template was initially processed with FreeSurfer v5.3 (Dale et al., 1999; Fischl et al., 1999) to generate the cortical gray matter labels of the four major brain lobes by combining regional labels from the Desikan-Killiany atlas (Desikan et al., 2006)^1^. The merged cortical labels for the four lobes were extended to the closest underlying WM and subcortical regions, delineating the volumetric regions of interest (ROI) of the four major lobes in MNI152 template space. The Greedy SyN algorithm from the Advanced Normalization Tools (ANTs; Avants et al., 2011) software was then used to non-linearly register the MNI152 template (McConnell Brain Imaging Centre, Montreal Neurological Institute, McGill University) to each participant’s T1 MRI scan. The resulting spatial warping parameters were then applied to the four lobes in each hemisphere to generate native space lobar ROIs for each participant. Finally, these native space lobar ROIs were used as search regions for the binarized lesion probability maps obtained from LST to identify regional WMH volumes from each hemisphere. To evaluate regional effects, lobar WMH values were obtained by summing the left and right hemisphere WMH volumes for the frontal, temporal, parietal, and occipital lobes. Total and lobar WMH volumes were natural log-transformed, as raw volumes were not normally distributed, and adjusted for total intracranial volume (TIV; Alexander et al., 2012) with linear regression.

### Statistical Analysis

Differences between the low and high PSA groups were evaluated using *t*-tests and chi-square tests, where appropriate. Two-way analysis of covariance (ANCOVA) was used to test the effects of age group (YO = 50–69 years vs. OO = 70–89 years), PSA group (low vs. high), and their interaction on total and lobar WMH volumes after we adjusted for the initial covariates of sex, hypertension status, and body mass index (BMI). ANCOVA models with significant two-way interactions were followed by simple effect analyses with the same covariates.

## Results

Participants who reported high levels of PSA (*n* = 36) were compared to those who reported low levels of PSA (*n* = 160). The two groups do not differ in age, years of education, MMSE score, hypertension status, and BMI (*p*’s > 0.05). Sex differed between the groups with more men in the high PSA group (*p* = 0.04). Table 1 presents participant characteristics for the two PSA groups.

**Table 1:** Subject characteristics (mean ± SD).

	Total	Low PSA	High PSA	*p*-values
*N*	196	160	36	-
Age (years)	69.76 ± 10.58	69.38 ± 10.92	71.45 ± 8.85	0.29
Sex (F/M)	95/101	83/77	12/24	0.04
Education (years)	15.83 ± 2.56	15.76 ± 2.63	16.17 ± 2.24	0.39
MMSE	28.96 ± 1.24	28.89 ± 1.29	29.28 ± 0.97	0.09
Hypertension status (%)	32.65	30.63	41.67	0.20
BMI	25.42 ± 3.98	25.40 ± 4.10	25.51 ± 3.45	0.89

*Note: PSA, physical sports activity; F, female; M, male; MMSE, Mini Mental State Exam; BMI, body mass index*.

### Total White Matter Hyperintensity Volume

**Figure 2:**
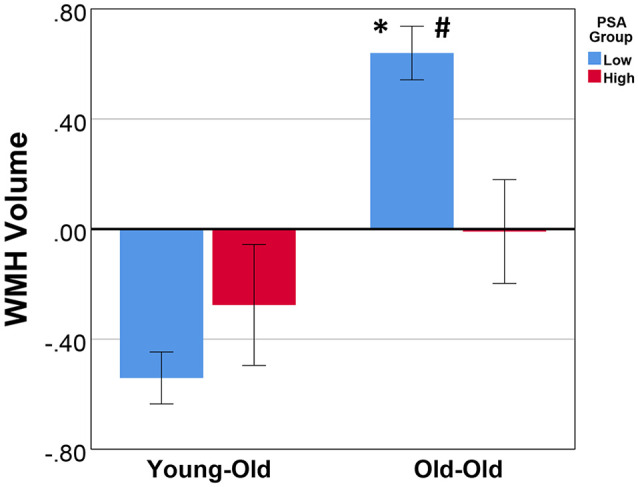
The mean and standard error of total WMH volume for age and physical sports activity groups. Analysis of covariance (ANCOVA) showed that, after controlling for sex, hypertension status, and body mass index, there was a significant main effect for age group (*p* = 1.23 E-5), no main effect for PSA group (*p* = 0.23), and a significant age group by PSA group interaction (*p* = 0.005). Simple effects revealed that: ^#^within the OO group, the low PSA had significantly greater WMH volume (*p* = 0.003); *within the low PSA group, the OO had significantly greater WMH volume (*p* = 2.37 E-15). There was no significant difference between the YO with low vs. high PSA (*p* = 0.27) and the YO vs. OO with high PSA (*p* = 0.36). WMH volumes were natural log-transformed and TIV adjusted. Additionally, all significant effects remained after an ANCOVA was performed on the total intracranial volume (TIV) adjusted total WMH volume without natural log transformation, *p* ≤ 0.009. WMH, white matter hyperintensity; PSA, physical sports activity; OO, old-old; YO, young-old.

A significant main effect for age group (*F*_(1,189)_ = 20.17, *p* = 1.23 E-5) was observed with the OO having greater WMH volume than the YO, but the main effect of the PSA group was not significant (*F*_(1,189)_ = 1.43, *p* = 0.23). There was a significant age group by PSA group interaction (*F*_(1,189)_ = 8.26, *p* = 0.005; Table 2). Follow-up simple effects analyses indicated that the OO group with low PSA had significantly greater WMH volume than both the OO with high PSA (*F*_(1,189)_ = 9.38, *p* = 0.003) and the YO with low PSA (*F*_(1,189)_ = 74.62, *p* = 2.37 E-15). In contrast, there were no significant differences between the YO and OO with high PSA (*F*_(1,189)_ = 0.85, *p* = 0.36) or between the YO with low PSA and the YO with high PSA (*F*_(1,189)_ = 1.23, *p* = 0.27; Figure 2). Additionally, all significant effects remained after ANCOVAs were performed on total WMH volume without natural log transformation, *p* ≤ 0.009.

**Table 2:** Age group and physical sports activity group differences in WMH volume.

	Two-way ANCOVA (Mean ± SE)				*p*-value		
	Low PSA		High PSA		PSA	Age	Age × PSA
WMH	Young-Old	Old-Old	Young-Old	Old-Old			
Total	−0.54 ± 0.09^E,I^	0.64 ± 0.10^A,E^	−0.28 ± 0.22^I,M^	−0.01 ± 0.19^A,M^	0.23	1.23E-5*	0.005*
Frontal	−0.58 ± 0.09^F,J^	0.63 ± 0.10^B,F^	−0.27 ± 0.22^J,N^	0.15 ± 0.19^B,N^	0.57	9.11E-7*	0.01*
Temporal	−0.48 ± 0.10^G,K^	0.56 ± 0.10^C,G^	−0.30 ± 0.22^K,O^	0.02 ± 0.19^C,O^	0.26	5.18E-5*	0.03*
Parietal	−0.52 ± 0.10^H,L^	0.62 ± 0.10^D,H^	−0.41 ± 0.22^L,P^	0.09 ± 0.19^D,P^	0.20	1.06E-6*	0.04*
Occipital	−0.29 ± 0.11	0.38 ± 0.11	−0.15 ± 0.25	−0.15 ± 0.21	0.28	0.07	0.06

Note: SE, standard error; PSA, physical sports activity; WMH, white matter hyperintensity; values after natural log transformation and adjusting for total intracranial volume (TIV) while controlling for sex, hypertension status, and body mass index. Simple effects analyses revealed the following (*p*-values): within the OO group, low and high PSA differed for total (*A* = 0.003), frontal (*B* = 0.02), temporal (*C* = 0.01), and parietal (*D* = 0.01) WMH volume; within the low PSA group, YO differed from OO for total (*E* = 2.37 E-15), frontal (*F* = 5.77 E-16), temporal (*G* = 4.14 E-12), and parietal (*H* = 1.94 E-14) WMH volume; within the YO group, low and high PSA did not differ for total (*I* = 0.27), frontal (*J* = 0.20), temporal (*K* = 0.48), and parietal (*L* = 0.63) WMH volume; within the high PSA group, YO did not differ from OO for total (*M* = 0.36), frontal (*N* = 0.15), temporal (*O* = 0.28), and parietal (*P* = 0.09) WMH volume; *significant at *p* ≤ 0.026 with ANCOVAs performed on WMH volumes without natural logarithmic transformation.

### Regional White Matter Hyperintensity Volume

#### Frontal Lobe Region

We found a significant main effect for age group (*F*_(1,189)_ = 25.79, *p* = 9.11 E-7) for frontal lobe WMH volume with the OO group having greater frontal WMH volume than the YO. There was no main effect for PSA group (*F*_(1,189)_ = 0.32, *p* = 0.57), but there was a significant age group by PSA group interaction (*F*_(1,189)_ = 6.15, *p* = 0.01). Simple effects analyses indicated that the OO with low PSA had significantly greater frontal WMH volume than both the OO with high PSA (*F*_(1,189)_ = 5.24, *p* = 0.02) and the YO with low PSA (*F*_(1,189)_ = 78.62, *p* = 5.77 E-16). There were no significant differences between the YO with low and high PSA (*F*_(1,189)_ = 1.62, *p* = 0.20) or between the YO and OO with high PSA (*F*_(1,189)_ = 2.14, *p* = 0.15). All significant effects remained after ANCOVAs were performed on frontal WMH volume without natural log transformation, *p* ≤ 0.013.

#### Temporal Lobe Region

We observed a significant main effect for age group (*F*_(1,189)_ = 17.17, *p* = 5.18 E-5) for temporal lobe WMH volume with the OO having greater WMH volume than the YO. There was no main effect for PSA group (*F*_(1,189)_ = 1.29, *p* = 0.26), but there was a significant age group by PSA group interaction (*F*_(1,189)_ = 4.91, *p* = 0.03). Follow-up simple effects analyses indicated that the OO with low PSA had significantly greater temporal WMH volume than both the OO with high PSA (*F*_(1,189)_ = 6.38, *p* = 0.01) and the YO with low PSA (*F*_(1,189)_ = 54.96, *p* = 4.14 E-12). Furthermore, there were no significant differences between the YO with low and high PSA (*F*_(1,189)_ = 0.51, *p* = 0.48) or between the YO and OO with high PSA (*F*_(1,189)_ = 1.19, *p* = 0.28). All significant effects remained after ANCOVAs were performed on temporal WMH volume without natural log transformation, *p* ≤ 0.026.

#### Parietal Lobe Region

There was a significant main effect for age group (*F*_(1,189)_ = 25.45, *p* = 1.06 E-6) for parietal lobe WMH volume. There was no main effect for PSA group (*F*_(1,189)_ = 1.66, *p* = 0.20), but a significant age group by PSA group interaction (*F*_(1,189)_ = 4.09, *p* = 0.04) was observed. Simple effects analyses indicated that the OO with low PSA had significantly greater parietal WMH volume than both the OO with high PSA (*F*_(1,189)_ = 6.22, *p* = 0.01) and the YO with low PSA (*F*_(1,189)_ = 68.89, *p* = 1.94 E-14). There were no significant differences between the YO with low and high PSA (*F*_(1,189)_ = 0.23, *p* = 0.63) or between the YO and OO with high PSA (*F*_(1,189)_ = 2.88, *p* = 0.09). All significant effects remained after ANCOVAs were performed on parietal WMH volume without natural log transformation, *p* ≤ 0.024.

#### Occipital Lobe Region

In contrast, there was a non-significant trend for the main effect of age group (*F*_(1,189)_ = 3.28, *p* = 0.07) and a non-significant trend for the age group by PSA group interaction (*F*_(1,189)_ = 3.52, *p* = 0.06) for occipital lobe WMH volume. There was no main effect for PSA group (*F*_(1,189)_ = 1.17, *p* = 0.28). All effects remained non-significant after an ANCOVA was performed on occipital WMH volume without natural log transformation, *p* ≥ 0.054.

## Discussion

In this cohort of community-dwelling, neurologically healthy older adults 50–89 years of age, we found that self-report of engagement in PSA interacts with age on WMH volume. Although older age was associated with greater total WMH volumes, this age difference was only observed in those who reported low PSA levels. In contrast, the YO and OO groups reporting high levels of PSA did not differ in total WMH volumes, supporting the potential benefits of PSA in reducing WM lesion load during aging. We also observed no difference between YO participants with low and high PSA which further suggests that the main impact of WMH volume accumulation in otherwise healthy older adults occurs to a greater extent in the eighth and ninth decades. As such, engaging in PSA may have its greatest benefits in reducing the risk for cerebrovascular disease associated with WM lesion load in this older age group.

These WM findings were also observed when we evaluated the effects of age and PSA on lobar regional WMH volumes, including the frontal, temporal, and parietal lobes. Although there was no significant age group by PSA group interaction in the occipital lobe, a trend was observed that followed the same pattern of the other regional WMH values. Previous studies have suggested that frontal WM may be preferentially vulnerable to the accumulation of WMH volumes with age (Holland et al., 2008; Marquine et al., 2010). Although the interactions of PSA with age group across the frontal, temporal, and parietal lobar regional WMH volumes in our sample were each significant, the relative comparison of their effect sizes appears consistent with a posterior to the anterior gradient for the modifying effects of PSA on age group in our healthy aging cohort. In this case, relative to the effect size of the PSA by age group interaction for parietal WMH volume ($\eta_{\text{p}}^{2}$ = 0.021), the interaction effect was 23.8% greater for temporal WMH volume ($\eta_{\text{p}}^{2}$ = 0.026) and 52.4% greater for the frontal WMH volume ($\eta_{\text{p}}^{2}$ = 0.032). Thus, our findings suggest that high levels of PSA may mitigate the impact of age on WMH volumes in those regions thought to be most vulnerable to brain aging effects.

Although more men reported engaging in high levels of PSA in our cohort, there were no significant differences between the low and high PSA groups in age, years of education, MMSE scores, hypertension status, or BMI. Furthermore, the effects of age and PSA were observed after we controlled for sex, hypertension status, and BMI for the total and regional WMH volumes. Together, these results suggest that engaging in high levels of PSA may help maintain WM in the context of healthy aging by reducing WM lesion load, especially in the frontal, temporal, and parietal lobe regions. It is important to note, however, that our findings cannot rule out the possibility that having higher levels of WMH might also contribute to less engagement in PSA. Follow up studies are needed to further evaluate the causal connections between PSA and WMH volume in aging.

Our findings are consistent with previous research on the relationship between PA and total WMH volume. A study by Saczynski et al. (2008) reported that older adults in the upper quartile of WM lesion load were significantly more likely than those in the lower three quartiles to be physically inactive. Additionally, in a sample of community-dwelling cognitively normal older adults, Wirth et al. (2014) reported that higher current PA was significantly related to lower WM lesion volume, which mediated the beneficial effects of current PA on WM integrity. Moreover, our findings support previous research on age-related increases in WMH volume, particularly in frontal regions. A study by Holland et al. (2008) found that WMH volume in healthy older adults was predominantly distributed around the frontal and posterior horns of the lateral ventricles as well as in the parietal lobe. Additionally, in a sample of patients with Alzheimer’s disease (AD) and mild cognitive impairment (MCI), as well as cognitively unimpaired, healthy elderly controls, Chen et al. (2006) reported that WMH volume was significantly correlated with age, particularly for frontal WMH volume.

The current study used self-report to assess high levels of sports activity, which have been suggested to provide a more reliable self-report measure of PA in older adults (Sylvia et al., 2014). Nevertheless, additional work using quantitative measures of MVPA with actigraphy is warranted to further evaluate the effects of different levels of PA on brain aging over the lifespan (Raichlen and Alexander, 2017; Raichlen et al., 2019). While sports activity has not been widely explored as a specific type of PA often performed by older adults, it is increasingly recognized as an important factor that can benefit cognitive aging and brain health. Recommendations by the WHO have highlighted the potential benefits for older adults to improve cardiorespiratory fitness and reduce the risks for heart disease, stroke, and cognitive decline by engaging in at least 150 min of moderately intense aerobic PA, 75 min of vigorously intense aerobic PA, or an equivalent combination of moderate- and vigorous-intensity activity throughout the week (World Health Organization, 2010). In the current study, engagement in high levels of PA corresponds with the WHO recommendations. Furthermore, a study by Awick et al. (2017) found that increases in MVPA among older adults were significantly associated with reductions in psychological distress which, in turn, was associated with improvements in quality of life. Thus, engagement in high levels of PA, such as sports activity, may provide beneficial effects that extend beyond improved brain health, including emotional and social well-being. This may be especially the case for those activities that often include social interactions, reflecting an area for further study.

Previous research on the potential effects of PSA on WMH volume have often not evaluated both total and regional volumes; have not considered interactive effects of age and PSA; have not consistently accounted for cardiovascular health factors, such as hypertension status and BMI; and often include clinical populations rather than community-dwelling healthy older adults. Our sample was mainly comprised of Caucasian participants and, as such, our findings may not apply to other racial or ethnic groups. The combined effects of age and PSA on total and regional WMH volumes should be further evaluated in larger, ethnically diverse samples. Also, by assessing self-reported PSA throughout the past year, we are unable to evaluate the impact of lifelong engagement in sports activity. Although some studies of WMH load in aging have distinguished between deep and periventricular lesions, our automated quantitative method is not able to identify volume differences for these spatially distinct WMH lesion subtypes. Future work to obtain quantitative segmentation of deep vs. periventricular WMH volumes is warranted to help evaluate potential differences in their relation to brain aging. There is also growing interest in understanding how WMH volumes interact with gray matter in healthy aging and with cerebrovascular risk factors, like hypertension (e.g., Kern et al., 2017). Future studies using multimodal neuroimaging methods are needed to evaluate how associations between WMH volume and gray or WM integrity are altered by PSA in the context of healthy and pathological aging. Moreover, our study of WMH volume and PSA was cross-sectional and, thus, limited to evaluating differences between age groups. Future research would benefit from assessing the role of longitudinal variability in PSA and its potential impact on brain health, as well as for different types of sports activities. Follow-up longitudinal assessments are also needed to investigate the temporal relation between WMH volume, PA level, and age to evaluate the impact of PSA over time in an otherwise healthy aging population. Also, experimental and intervention studies of PSA are needed to help clarify the causal relation and associated mechanisms for engagement in leisure sports activities and WMH in aging. Lastly, longitudinal studies are needed to determine if enhanced brain health, as measured by reduced WMH volumes, following engagement in aerobic PSA helps to decrease the risk for future cognitive decline and the development of age-related neurodegenerative disorders, like AD (Alexander, 2017).

## Conclusions

Increases in WMH volumes have been associated with brain aging and cognitive decline (Tseng et al., 2013; Prins and Scheltens, 2015), as well as increases in risk for cerebrovascular disease, AD (Lee et al., 2008; Alexander, 2017), and mortality (Debette and Markus, 2010). Our findings suggest that high levels of self-reported PSA modify the association between increasing age and greater WMH volumes, which may help to diminish both the total and regional distribution of WMH volumes often observed in older adults. Engagement in high levels of PSA may be an important modifiable lifestyle factor that can potentially help to maintain brain health in older age, especially in regions that tend to be most vulnerable to healthy aging effects, such as in the frontal lobes. Future work is needed to further evaluate the potential of aerobic PSA as a lifestyle intervention to alter the trajectory of cognitive decline in healthy and pathological aging, potentially reducing the risk for AD and other age-related neurodegenerative diseases.

## Data Availability Statement

The raw data supporting the conclusions of this article are made available by the authors, without undue reservation.

## Ethics Statement

The studies involving human participants were reviewed and approved by the University of Arizona IRB Committee. The participants provided their written informed consent to participate in this study.

## Author Contributions

MF, YK, and DR contributed to data analyses, interpretation, and manuscript preparation. PB contributed to data preparation, analyses, interpretation, and manuscript preparation. LN contributed to study recruitment, data collection, and data preparation. EV contributed to data analyses and manuscript preparation. GH and TT contributed to data collection, data preparation, interpretation, and manuscript preparation. GA contributed to study oversight, study recruitment, data analyses, interpretation, and manuscript preparation.

## Conflict of Interest

The authors declare that the research was conducted in the absence of any commercial or financial relationships that could be construed as a potential conflict of interest.

## Footnotes

^1^https://surfer.nmr.mgh.harvard.edu/fswiki/CorticalParcellation
